# snoRNA, a Novel Precursor of microRNA in *Giardia lamblia*

**DOI:** 10.1371/journal.ppat.1000224

**Published:** 2008-11-28

**Authors:** Ashesh A. Saraiya, Ching C. Wang

**Affiliations:** Department of Pharmaceutical Chemistry, University of California San Francisco, San Francisco, California, United States of America; Washington University School of Medicine, United States of America

## Abstract

An Argonaute homolog and a functional Dicer have been identified in the ancient eukaryote *Giardia lamblia*, which apparently lacks the ability to perform RNA interference (RNAi). The *Giardia* Argonaute plays an essential role in growth and is capable of binding specifically to the m^7^G-cap, suggesting a potential involvement in microRNA (miRNA)-mediated translational repression. To test such a possibility, small RNAs were isolated from *Giardia* trophozoites, cloned, and sequenced. A 26-nucleotide (nt) small RNA (miR2) was identified as a product of Dicer-processed snoRNA GlsR17 and localized to the cytoplasm by fluorescence *in situ* hybridization, whereas GlsR17 was found primarily in the nucleolus of only one of the two nuclei in *Giardia*. Three other small RNAs were also identified as products of snoRNAs, suggesting that the latter could be novel precursors of miRNAs in *Giardia*. Putative miR2 target sites were identified at the 3′-untranslated regions (UTR) of 22 variant surface protein mRNAs using the miRanda program. *In vivo* expression of *Renilla* luciferase mRNA containing six identical miR2 target sites in the 3′-UTR was reduced by 40% when co-transfected with synthetic miR2, while the level of luciferase mRNA remained unaffected. Thus, miR2 likely affects translation but not mRNA stability. This repression, however, was not observed when Argonaute was knocked down in *Giardia* using a ribozyme-antisense RNA. Instead, an enhancement of luciferase expression was observed, suggesting a loss of endogenous miR2-mediated repression when this protein is depleted. Additionally, the level of miR2 was significantly reduced when Dicer was knocked down. In all, the evidence indicates the presence of a snoRNA-derived miRNA-mediated translational repression in *Giardia*.

## Author Summary

Gene regulation in *Giardia lamblia*, a primitive parasitic protozoan responsible for the diarrheal disease giardiasis, is poorly understood. There is no consensus promoter sequence. A simple eight–base pair AT-rich region is sufficient to initiate gene transcription in this organism. Thus, the main control of gene expression may occur after the stage of transcription. The presence of Dicer and Argonaute homologs in *Giardia* suggested that microRNA (miRNA)-mediated translational repression could be one mechanism of gene regulation. In this work, we characterized the presence of the miRNA pathway in *Giardia* as well as identified the novel use of small nucleolar RNA (snoRNA) as miRNA precursors. Potential target sites for one small RNA (miR2) were identified with the miRanda program. *In vivo* reporter assays confirmed the specific interaction between the target sites and miR2. A ribozyme-mediated reduction of Dicer and Argonaute in *Giardia* showed that the former is required for miR2 production whereas the latter functions in mediating the inhibition of reporter expression, which agrees with the roles of these two proteins. This is the first evidence of miRNA-mediated gene regulation in *Giardia* and the first demonstration of the use of snoRNAs as miRNA precursors.

## Introduction

The role of small non-coding RNAs in gene regulation has been extensively studied in recent years [1]. MicroRNAs (miRNA) are a major class of small RNAs that are involved in gene regulation via a translational repression mechanism. They play important roles in regulation of genes involved in development [2], cell differentiation [3], and cell maintenance [4]. In higher eukaryotes, maturation of miRNAs from the initial RNA Polymerase II transcripts requires the actions of several proteins. Drosha, a nuclear endoribonuclease III, is known to cleave the primary-miRNAs to produce pre-miRNAs [5]. Exportin5 is responsible for exporting the pre-miRNAs out of the nucleus [6],[7]. Dicer, a cytoplamic endoribonuclease III, cleaves the pre-miRNAs to produce mature miRNAs [8]. Argonaute, which is a major component in the RNA-induced silencing complex (RISC), binds to the mature miRNA [9],[10]. An imperfect complementation between the miRNA incorporated into the RISC complex and its target site located at the 3′-untranslated region (UTR) of mRNA results in translational repression [11],[12]. One possible mechanism of repression involves binding of the Argonaute in the RISC complex to the 7-methylguanosine (m^7^G) cap of the mRNA resulting in inhibition of translation initiation [13].

*Giardia lamblia* is a unicellular and binucleated protozoan responsible for giardiasis in humans [14]. Phylogenetic analysis has classified it as one of the earliest branching eukaryotes with many primitive features [14]. In higher eukaryotes, gene expression is highly regulated both transcriptionally and translationally. In *Giardia*, however, few consensus promoters have been identified and an 8 bp AT-rich region was sufficient to initiate transcription [15]. Additionally, *Giardia* mRNAs have exceedingly short 3′ and 5′-UTRs, thus greatly reducing the availability of regulatory sites for translational regulation. For instance, ribosomal scanning, an essential mechanism for translation initiation in higher eukaryotes and yeast, is absent from *Giardia* [16]. Therefore, *Giardia* represents a unique model for studying the evolution of eukaryotic translational regulation.

No RNA interference (RNAi) has been identified in *Giardia* in spite of repeated trials by several laboratories in the past (data unpublished). An analysis of the *Giardia* genome showed no homolog of Drosha or Exportin5. It, however, identified a Dicer (XP 001705536) and an Argonaute homolog (XP 001707926). *Giardia* Dicer is the only Dicer protein whose three-dimensional structure has been resolved by X-ray crystallography [17]. It was shown to cleave double-stranded RNA (dsRNA) *in vitro* and support RNAi in a *Schizosaccharomyces pombe* Dicer deletion mutant [17]. *Giardia* Dicer is thus likely a *bona fide* functional Dicer. Though the function of *Giardia* Argonaute-like protein (GlAgo) remains largely unexplored, antisense-ribozyme-mediated knockdown of this protein inhibited cell growth and purified recombinant GlAgo can bind specifically to m^7^G-cap-Sepharose (see below). These data raised the possibility that the Argonaute and Dicer in *Giardia* may be involved in miRNA-mediated translational repression.

Abundant antisense RNAs, up to 20% of the total mRNAs, and small RNAs have been identified in *Giardia* [18],[19]. About 20 snoRNAs have also been identified in *Giardia* [20]. Previous efforts at cloning small RNAs from *Giardia* have resulted in identifying small RNAs homologous to the telomeric retroposons, which were postulated to function in silencing retroposons [19]. In our current study, we isolated, cloned and sequenced small RNAs from *Giardia* and identified some of the known snoRNA sequences among them. One of the small RNAs, miR2, was identified as a Dicer-digested product from GlsR17, previously identified as a box C/D snoRNA in *Giardia* [20]. Putative target sites for miR2 were identified at the 3′-UTRs of many variant surface protein (VSP) mRNAs. Expression of a reporter mRNA carrying these putative target sites was specifically inhibited by miR2 without affecting the mRNA level. Subsequent analysis also indicated the dependence of this inhibition on the presence of Argonaute, thus verifying the ability of a snoRNA derived miRNA to function in miRNA-mediated translational repression in *Giardia*.

## Results

### The Effect of Knocking Down Argonaute on *Giardia* Growth

To find out if GlAgo plays a critical role in the proliferation of *Giardia*, mRNA encoding this protein was knocked down by expressing an antisense-ribozyme RNA in giardiavirus-infected *Giardia* trophozoites [21]. Quantitative RT-PCR indicated that the mRNA was reduced by 50% in the transfected cells (data not shown). Growth of the knockdown cells was inhibited, reaching only 58% of the wild type level after 4 days of cultivation (Figure S1A), suggesting that GlAgo plays an important role in *Giardia* growth.

### Binding of Recombinant Argonaute to m^7^G-cap-Sepharose

Recent studies of human Argonaute have identified the presence of a cap-binding motif in the protein [13], which enables it to compete with eIF4E for binding to the m^7^G cap in mRNA that may explain a part of the mechanism involving Argonaute in miRNA-mediated translational repression [13]. To test whether this cap-binding motif is also present in GlAgo, m^7^G-Sepharose was incubated with recombinant GlAgo purified from *E. coli*. The majority of the GlAgo was found in the flow-through suggesting an excessive loading of the recombinant protein to the beads. After extensive washing with the binding buffer and buffer containing 0.1 mM GTP to remove non-specific binding, the protein was specifically eluted off the Sepharose beads with m^7^GpppG (Figure S2), suggesting that GlAgo binds to the m^7^G cap in a highly specific manner, which has been demonstrated to be the cap of *Giardia* mRNA [22]. Therefore, GlAgo may function in miRNA-mediated translational repression in *Giardia* by competing with eIF4E for binding to the mRNA m^7^G-cap [13].

### Identification of Putative miRNAs in *Giardia*

To verify if miRNA-mediated translational repression is functional in *Giardia*, total RNA was isolated from cultures of *Giardia* WB trophozoites and size fractionated for small RNAs of <40 nucleotides (nts). Isolated small RNAs were cloned using RNA linkers that require the presence of a 5′-phosphate in the small RNA to be ligated by T4 RNA ligase [23]. Therefore, all the cloned small RNAs should contain a 5′ phosphate, which is a known characteristic of Dicer processing product [23]. A library of 101 clones with unique sequences ranging in sizes between 20 and 34 nts was created with the following distributions: 3% 20 nts, 11% 21 nts, 12% 22 nts, 8% 23 nts, 6% 24 nts, 7% 25 nts, 14% 26 nts, 6% 27 nts, 5% 28 nts, 5% 29 nts, 9% 30 nts, 9% 31 nts, 5% 32 nts, and 1% 34 nts. Interestingly, the size distribution results in a peak at 26 nts, which is the expected size of *Giardia* Dicer cleaved products based on the crystal structure [17]. Of these sequences, 11 were found to be identical to other clones in the library but with longer nucleotide extensions at the 3′ ends. The shorter RNAs were presumed to be degradation products and were discarded. Potential origins of the small RNAs were identified by BLAST search analysis of the *Giardia* genome database [24]. The results showed that 81 of the 101 clones were fragments of ribosomal RNA or tRNA and were also discarded. Of the remaining clones, 15 were fragments of open reading frames, 4 were fragments of 3 different box C/D snoRNAs [20], and 1 was a fragment of retrotransposon GilT.

**Figure 1:**
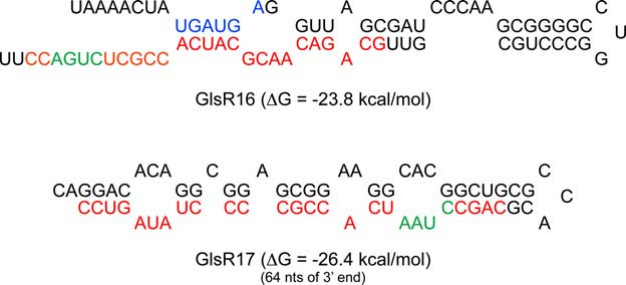
MFOLD analysis of snoRNAs GlsR16 and GlsR17. The sequences of mature snoRNA GlsR16 and the 64 nt 3′-portion of GlsR17 were submitted to the MFOLD RNA server. Both snoRNAs produced secondary structures that are suitable for being Dicer substrates. The red letters represent miR3 in GlsR16 and miR2 in GlsR17. The Box C (blue) and Box D (green) motifs are indicated. Both miRNAs are located at the 3′-end of their precursors.

Of the four small RNAs from snoRNAs, one is a 24 nt fragment (miR4) from GlsR1 (85 nts), two are 21 and 26 nt 3′-fragments (miR1 and miR3) of GlsR16 (77 nts) and one is a 26 nt 3′-fragment (miR2) from GlsR17 (144 nts). These non-coding RNAs have been previously described as snoRNA based on the presence of box C/D motifs [20] (see Discussion). GlsR1 is predicted to function in 2′-O-ribose methylation based on its sequence complementary to that of *Giardia* 16S ribosomal RNA as well as its homology to yeast snR70 [20]. SnoRNAs GlsR17 and GlsR16, however, did not contain antisense sequences complementary to ribosomal RNA and were considered to be “orphan” snoRNAs. Therefore, miR2 (5′ CAG CCU AAU CAC CGC CCC UAU AGU CC 3′) from snoRNA GlsR17 and miR1 (5′ CAA CGC ATC ACC GCT CTG ACC 3′) and miR3 (5′ GCA GAC AAC GCA TCA CCG CTC TGA CC 3′) from snoRNA GlsR16 were of particular interest in terms of their potential function in *Giardia*. Box C/D snoRNAs typically form a hairpin structure with the 5′ and 3′ ends forming part of the stem. Therefore, it is not surprising that MFOLD analysis of full-length snoRNA GlsR16 and the 64 nt 3′-portion of GlsR17 resulted in the formation of thermodynamically favorable hairpin loop structures with the corresponding miRNAs localized to one of the two arms at the 3′-end (Figure 1) [25]. Interestingly, MFOLD analysis of the full length GlsR17 resulted in a double stem loop structure similar to that observed for box H/ACA snoRNAs with miR2 located in the second hairpin structure (Figure S3). The relatively small sizes and the hairpin loop structures of these snoRNAs qualify them as suitable substrates for Dicer action.

**Figure 2:**
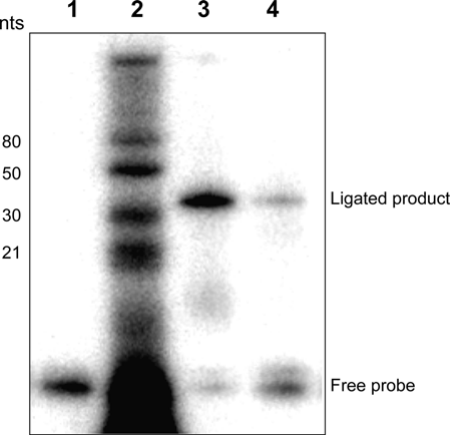
Splinted ligation of miR2. Size fractionated RNA (<40 nts) was analyzed by splinted ligation for the presence of miR2. (Lane 1) Negative control containing only the labeled RNA probe. (Lane 2) RNA markers. (Lane 3) Positive control using a synthetic miR2. (Lane 4) Size fractionated RNA (200 ng) from *Giardia* WB trophozoites. The presence of a discrete band migrating to the same position with the positive control suggests the presence of miR2 in the small RNA population.

To ascertain that miR2 and miR3 are not random degradation products isolated during the initial cloning of small RNAs, their presence in the RNA from *Giardia* was monitored. The presence of GlsR16 and GlsR17 in total *Giardia* RNA was repeatedly demonstrated by Northern analysis as anticipated (data not shown). But miR2 and miR3 remained undetectable on these blots presumably due to their relatively low levels. A splinted ligation analysis was then performed on the size-fractionated small RNA samples (<40 nts) because of its relatively high sensitivity based on direct labeling of the specific small RNA of interest through a 3′-end specific ligation [26]. The results from this analysis confirmed the presence of miR2 in the original small RNA sample (Figure 2). The single labeled band with the anticipated size indicates that miR2 is a product of precise processing of GlsR17 in *Giardia* and not the result of nonspecific degradation. Splinted ligation analysis of miR3 also showed a single specific band with the anticipated size of a 26 nt RNA but not a 21 nt RNA (data not shown). Thus, miR3 is also a likely natural product from GlsR16 in *Giardia* whereas miR1 is more likely a nonspecific degradation product. A similar analysis of the small RNA derived from retrotransposon GilT failed to produce a specifically labeled band (data not shown). These data indicate that miR2 and miR3 are naturally processed small RNAs from their respective snoRNAs while the retrotransposon GilT small RNA is probably a degradation product.

### Identification of the Putative miR2 Target Sites in the 3′-UTRs of mRNA

Identification of putative miR2 target sites in the *Giardia* genome was performed using the miRanda program [27]. Since most known miRNA target sites have been localized to the 3′- UTRs of mRNAs [28], we focused our target identification to the 3′-UTRs of *Giardia* open reading frames (ORF). To limit the number of possible candidate target sites, segments of 250 nts (50 nts upstream and 200 nts downstream from the stop codon) were extracted from each of the 9,649 ORFs in the *Giardia* genome database [24] and used to identify possible target sites for miR2 using miRanda (score threshold = 120, energy threshold = 20 kcal/mol, and scaling factor = 4). Identification was based on localization of the putative binding site (between 50 nts upstream and 50 nts downstream from the stop codon) and the presence of a seed sequence (5 consecutive nts, without G:U base pairings, within 3 nts of the 5′ end of the miRNA).

Of the 9,649 ORFs in the *Giardia* database [24], 296 ORFs contained potential target sites for miR2 based on the criteria mentioned above. Among these ORFs, 191 were hypothetical proteins and 105 were annotated proteins. Of the latter, 22 were variant-specific surface proteins (VSP) and 7 were trophozoite cysteine-rich surface antigens. VSPs are known to have a highly conserved 3′ end [29]. An alignment of 10 randomly chosen VSP sequences indicated that the last 100 nts at the 3′-end are highly conserved (data not shown). The predicted miR2 target site is 30 nts long with a 20 nt segment within the coding region and 10 nts in the 3′-UTR with the required perfect complementation between the seed sequence of miR2 and the target site located in the 3′-UTR. This remarkable conservation of a 3′-UTR sequence among the 22 VSP transcripts could mean that their expression is subject to a common mechanism of regulation mediated by miR2.

### Effect of miR2 on Translation of mRNA Carrying the Putative Target Site at 3′-UTR

**Figure 3:**
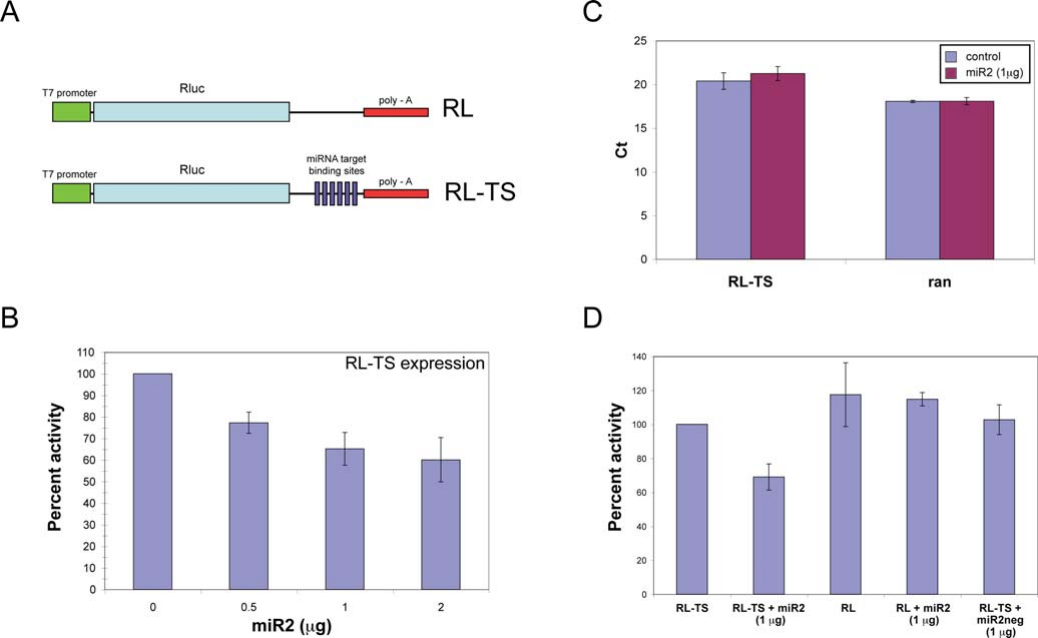
miR2-mediated translational repression of mRNA with putative target site at the 3′-UTR. (A) Diagram of the reporter constructs RL and RL-TS. (B) Percent luciferase activity of *Giardia* trophozoites transfected with RL-TS plus different concentrations of exogenous miR2. These results represent the average number and standard deviation of at least two replicates. (C) Real time quantitative RT-PCR comparison of RL-TS mRNA concentrations in the presence and absence of exogenously introduced miR2. Ran mRNA was monitored as a control. The data represent the average number and standard deviation from three independent transfection experiments. (D) Percent luciferase activity in RL-TS and RL transfected cells. The introduction of miR2 (1.0 µg) represses RL-TS activity by 35%. The absence of target sites (TS) in RL results in a small increase of expression by ∼15% that is not repressed by co-transfection with miR2. This suggests that the target sites are required for repression of luciferase activity by both endogenous and exogenous miR2. The use of a mutant miR2, miR2neg, does not affect the luciferase activity, indicating a specificity between miR2 and its target sites. These data represent the average number and standard deviation of four independent transfection experiments.

To test the potential consequence of *in vivo* interactions between miR2 and the putative target sites identified in the 3′-UTR of VSP transcripts, six copies of the putative binding sites from VSP AS12 were added to the 3′-UTR of *Renilla* luciferase gene in a plasmid construct (Figure 3A). Capped mRNA of this chimeric gene was transcribed *in vitro* (RL-TS) and electroporated into *Giardia* WB trophozoites together with chemically synthesized miR2. After incubation at 37°C for 5 hrs, the transformed *Giardia* cells were lysed and assayed for luciferase activity. Expression of RL-TS, when introduced into *Giardia* alone, was set at 100%. The inclusion of 0.5 µg of miR2 reduced the luciferase activity by 23% (Figure 3B), while 1 µg of miR2 decreased luciferase activity by 35% (Figure 3B). Little additional inhibition was observed when the concentration of miR2 was increased to 2 µg (40%) (Figure 3B). Thus, exogenously introduced miR2 does repress the expression of RL-TS in *Giardia* in a concentration-dependent but saturable manner. Quantitative RT-PCR estimation of the RL-TS mRNA levels in transfected *Giardia* with or without the presence of exogenously introduced miR2 resulted in two C_t_ values of 20.4±0.95 and 21.2±0.8, respectively (Figure 3C). This lack of apparent difference in mRNA levels indicates that the miR2 repression of luciferase expression is not due to enhanced mRNA degradation.

To confirm that the reduced luciferase activity is attributed to a specific interaction between miR2 and the putative target sites in the 3′-UTR of RL-TS, capped luciferase mRNA without the target sites (RL) was transfected into *Giardia* cells with or without 1 µg exogenous miR2. The luciferase activity increased in both cases to ∼115% of the control value (Figure 3D), suggesting that the repressive effect of miR2 requires the presence of target sites in the mRNA. It also suggests that the absence of target sites in the mRNA may also relieve the latter from repression by endogenous miR2 so that the expression of RL goes ∼15% beyond that of RL-TS (Figure 3D).

To further test the specificity of the interaction between miR2 and its target sites, *Giardia* cells were transfected with a mutant miR2, miR2neg (5′ p-CAG GGA UUA CAC CGC CCC UAU AGU CC 3′), in which the five underlined nucleotides of the “seed” sequence in miR2 were mutated to the complementary sequence. The miR2neg is not expected to interact with the target site. Luciferase activity of the RL-TS mRNA was not affected when it was introduced into *Giardia* with 1 µg of miR2neg. This suggests that the interaction between miR2 and its target sites is sequence specific and essential for the repression of luciferase activity (Figure 3D).

### Localization of Endogenous miR2

**Figure 4:**
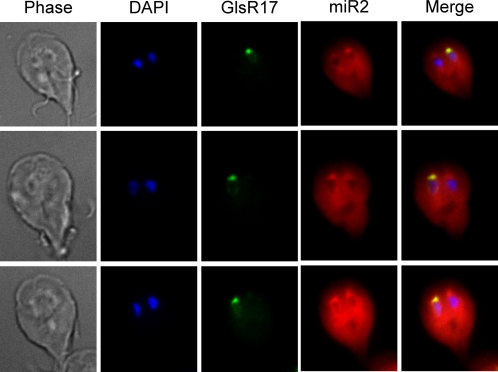
Localization of GlsR17 and miR2 in *Giardia* using FISH. GlsR17 is predominately localized to a single nucleus with a focus in the putative nucleolus in each *Giardia* trophozoite. miR2 is localized primarily to the cytoplasm.

snoRNAs are essential for the maturation of ribosomal RNA and are localized to the nucleolus. miRNAs, however, are localized to the cytoplasm to regulate gene expression. To verify the localizations of GlsR17 and miR2, fluorescence *in situ* hybridization (FISH) was performed. A 26 nt RNA probe directed against the 5′ end of GlsR17 was 5′-end labeled with FAM and another 26 nt probe complementary to miR2 at the 3′-end of GlsR17 was 5′ end labeled with Cy3. Hybridization of these probes to *Giardia* WB trophozoites resulted in an exclusive localization of GlsR17 to the nucleus with a specific focus in the putative nucleolus (Figure 4). This outcome is in good agreement with our previous finding that the trimethyl cap, known to cap the snoRNAs in *Giardia* [30], was specifically localized to the nucleolus-like organelle in *Giardia* nucleus [30]. The probe for the 3′-end of GlsR17, where miR2 is positioned, stained the nucleolus like the GlsR17 5′ probe, but the majority of the stain was found in the cytoplasm (Figure 4), indicating that miR2, the presumed product from GlsR17 by Dicer digestion, is localized primarily to the cytoplasm. Thus, in the apparent absence of Drosha and Exportin5, the 144 nt snoRNA GlsR17 could be transported into the cytoplasm and trimmed to the mature 26 nt miR2 by Dicer in *Giardia*.

A surprising and unexpected result from the FISH experiments was that the majority of nucleolar GlsR17 was found in only one of the two *Giardia* nuclei (Figure 4). While many previous studies indicate that the two nuclei in *Giardia* are virtually identical in many aspects [31],[32] (see Discussion), our current serendipitous finding may indicate functional differences related to snoRNAs between the two nuclei.

### *Giardia* Dicer

**Figure 5:**
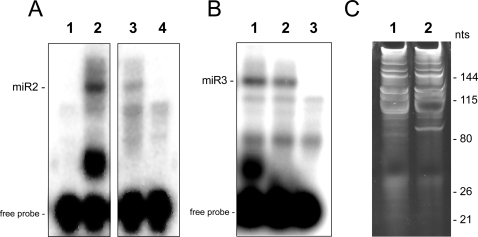
Effects of Dicer knockdown on levels of miR2 and miR3 in *Giardia* monitored by splinted ligation. (A) Lane 1, pegative control. Lane 2, positive control using a synthetic sample of miR2. Lane 3, a 33 ng sample of size fractionated small RNA (<40 nts) from giardiavirus-infected *Giardia* WB trophozoites transfected with an empty vector. Lane 4, a 33 ng sample of size fractionated small RNA (<40 nts) from giardiavirus-infected *Giardia* WB trophozoites transfected with the Dicer ribozyme. (B) Lane 1, positive control using a sample of synthetic miR3. Lane 2, a 33 ng sample of size fractionated small RNA (<40 nts) from giardiavirus-infected *Giardia* WB trophozoites transfected with an empty vector. Lane 3, a 33 ng sample of size fractionated small RNA (<40 nts) from giardiavirus-infected *Giardia* WB trophozoites transfected with the Dicer ribozyme. (C) Denatured PAGE analysis of size-fractionated RNA (<200 nts) stained with SYBR Gold. Lane 1, control cells; lane 2, dicer knockdown cells.

Dicer is responsible for the final maturation of miRNAs. We reasoned that if miR2 and miR3 were miRNAs involved in translational repression in *Giardia*, prior Dicer processing of their precursors would be required for their maturation. Therefore, a reduction in the Dicer level in *Giardia* would decrease the levels of both miR2 and miR3. For miR2, Dicer depletion would also relieve the repression of RL-TS expression by the endogenous miR2. Introduction of a specific antisense-hammerhead ribozyme RNA into giardiavirus-infected *Giardia* trophozoites was used to knock down *Giardia* Dicer mRNA [21]. This resulted in a 60% knockdown of Dicer mRNA as measured by semi-quantitative RT-PCR and an ∼47% decrease in the growth of *Giardia* after 4 days, indicating a role of Dicer in *Giardia* proliferation (Figure S1C and S1D). Splinted ligation analysis of size fractionated small RNAs (<40 nts) from Dicer knockdown cells showed a drastic decrease in the levels of both miR2 and miR3 (Figure 5A and 5B), indicating that *Giardia* Dicer is required to produce mature miR2 and miR3. To ascertain that other RNA species are not nonspecifically affected by the Dicer knockdown, size-fractionated RNAs (<200 nts) from the Dicer knockdown and the control cells were compared (Figure 5C). There is little difference among the RNAs with >100 nts between the two cell lines. But a band of ∼85 nts appears enhanced and a smeared band close to the 26 nt region seems diminished in the Dicer knockdown cells, which could represent certain miRNA precursors and total miRNAs, respectively. Dicer knockdown thus does not appear to affect RNA level nonspecifically.

Expression of capped RL-TS mRNA transfected into Dicer knockdown cells by electroporation showed a 15% increase from the wild type control (data not shown). This suggests that the lowered miR2 concentration, resulting from the Dicer knockdown, relieves the previously observed endogenous translational repression of RL-TS (Figure 3D).

### *Giardia* Argonaute

Domain analysis of GlAgo identified a PIWI domain but not a PAZ domain [33],[34]. Sequence alignments between GlAgo and the Argonautes of various origins, however, was able to identify both a PIWI and a PAZ domain [35]. Since the PAZ domain is known to interact with the small RNAs [36],[37], its apparent absence from GlAgo domain analysis raised the question whether GlAgo could bind to miR2. Recombinant His-tagged GlAgo was expressed and purified from transformed *Escherichia coli*. Gel shift analysis, using radiolabeled miR2 and recombinant GlAgo, indicated a slower moving radiolabeled band suggesting the formation of a GlAgo-miR2 complex (Figure S4, lanes 2 and 3). This binding of miR2 to GlAgo could be efficiently competed off by unlabeled miR2 (Figure S4, lane 5). Yeast RNA (Figure S4, lane 4) was also able to compete off miR2, indicating a lack of RNA sequence specificity for the binding. This result agrees with the previous observations on binding properties of Argonautes, which indicated that only the presence of a 5′ phosphate and a 3′ hydroxyl in an RNA molecule are required for binding but not sequence specificity [36]–[38]. Thus, GlAgo is apparently capable of binding small RNAs in the same way as the other Argonautes.

### Effect of an Argonaute Knockdown

**Figure 6:**
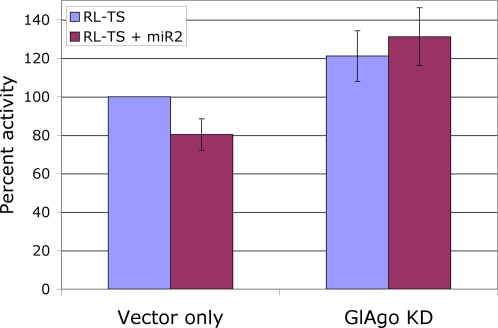
Loss of miR2 repression in GlAgo knockdown *Giardia* trophozoites. The translation of RL-TS in *Giardia* cells is repressed by introducing exogenous miR2 (1 µg) into the cells but not in the cells where GlAgo is partially knocked down. KD, knockdown.

To confirm that the repression of luciferase expression by miR2 in *Giardia* requires GlAgo, the GlAgo knockdown cells developed previously were transfected with the RL-TS transcript along with or without synthetic miR2. The results indicated that, in the absence of 50% of the GlAgo mRNA, expression of the luciferase activity exceeds that of the wild type control by about 20% (Figure 6). This enhanced activity was not affected by introducing exogenous miR2 into the cells, indicating that, by knocking down GlAgo, the repression of luciferase expression by both endogenous and exogenous miR2 was largely abolished. Thus, GlAgo is clearly playing an essential role in the miR2-mediated repression of luciferase expression in *Giardia*.

## Discussion

In the present study, we have provided evidence that (1) a miRNA-mediated mechanism of translational repression is present and functional in one of the most ancient eukaryotes *G. lamblia*; (2) the snoRNAs in *Giardia* can be precursors of miRNAs. To date, miRNA precursors have been identified among non-coding cellular transcripts [23],[39], 3′-UTRs of mRNAs [40],[41], introns [42], transposable elements [43], and viral transcripts [44]. Our current observation that snoRNAs can also function as a miRNA precursor, albeit in a deeply branched organism, has broadened the potential biological roles of this family of small RNAs and provided interesting speculations on the probable route of their evolution.

### snoRNAs

In eukaryotes, snoRNAs are generally regarded as being responsible for the maturation and modification of ribosomal RNA. Two families of snoRNAs have been identified based on sequence conservation, box C/D and box H/ACA. Box C/D snoRNAs contain conserved Box C (UGAUGA) and Box D (CUGA) elements near the 5′ and 3′ ends, respectively as well as internal copies of these elements termed Box C′ and Box D′ [45],[46]. An interaction between the 5′ and 3′ termini allows the formation of a stem bringing the Box C and Box D elements together to form a hairpin structure. Box C/D snoRNAs serve as the guide for 2′-O-ribose methylation of ribosomal RNA. Box H/ACA snoRNAs have secondary structures consisting of two hairpins separated by a hinge region and a short tail. The box H (ANANNA) element is located in the hinge region while an ACA element is located in the tail, 3 nucleotides from the 3′ end [45],[46]. Box H/ACA snoRNAs guide the pseudouridylation of ribosomal RNA. Interestingly, snoRNAs containing either the box C/D or box H/ACA motif but without an rRNA antisense region to guide modification of rRNA have been also identified [46]. The role of these “orphan” snoRNAs remains unclear. Dicer could theoretically trim the general hairpin structures of snoRNAs, which range from 60 to 160 nts in length in *Giardia*, without prior processing to produce potentially functional miRNAs.

In *Giardia*, small nuclear RNAs (snRNAs) were originally identified with antibodies directed against the trimethyl cap [47]. Subsequently, cloning and bioinformatics analysis identified approximately 20 characterized snoRNAs and 60 putative snoRNAs [20],[48]. GlsR16 and GlsR17 were among the 20 characterized snoRNAs and categorized in the orphan group. The 60 putative snoRNAs have not yet been tested experimentally for their real functions in *Giardia*, though some of them were postulated to modify rRNA due to the presence of anti-sense sequences that target rRNA. It is possible that some or all of them may function as precursors of miRNAs for specific translational repression. This is not to say that the snoRNAs are the only miRNA precursors in *Giardia*. Other non-coding RNAs, such as the abundant antisense RNAs [18], could also be a potential source for miRNA. However, in the apparent absence of Drosha/Pasha and Exportin5, one has to question the ability of *Giardia* Dicer to digest relatively large RNA hairpins. Giardiavirus, a dsRNA virus with a genome size of 6,277 bps, is known to multiply vigorously in the cytoplasm of infected *Giardia* trophozoites [49],[50]. This fact appears to rule out the possibility that *Giardia* Dicer could digest relatively long dsRNA. The snoRNAs identified in *Giardia* have lengths ranging from 60 to 160 nts [48], which appear to be the right size of RNA to fold into hairpin structures suitable for digestion by Dicer. Thus, the mature snoRNA could be a Dicer substrate without additional processing. So, it is not entirely unlikely that snoRNAs and perhaps some other small RNAs may constitute the reservoir for miRNA in *Giardia*. They could be exported from the nucleus by some yet unidentified means and digested by the Dicer for miRNA. Future investigations will provide answer to these intriguing possibilities.

### A Novel Mechanism for Pre-miRNA Production and the miRNA Pathway

In light of *Giardia's* minimalistic nature, it is reasonable to hypothesize that *Giardia* utilizes a single RNA processing mechanism for the maturation of both snoRNAs and miRNA precursors with characteristics similar to snoRNA. In human cells, snoRNAs are transcribed by RNA pol II [51]. Maturation of snoRNAs from the primary transcript involves processing of the 3′ end and hypermethylation of the m^7^G cap to a trimethyl cap (m^2,2,7^G) [51]. Recently, studies have shown that snoRNAs have a cytoplamic component during biogenesis. snoRNAs injected into the cytoplasm of *Xenopus* oocytes are imported into the nucleus, indicating a mechanism for nuclear transport [52]. Additionally, snoRNAs have been shown to associate with the nuclear export complex [53],[54] and reach their maturation in the cytoplasm [55]. Although this mechanism has only been shown for snoRNAs known to be involved in rRNA maturation, it is possible that “orphan” snoRNAs may also mature utilizing the same mechanism.

The outcome from our FISH experiments indicated the localization of GlsR17 primarily in the nucleolus and miR2 in the cytoplasm (Figure 4). A faint signal of GlsR17 was, however, also detectable in the cytoplasm. It is unclear if this signal is due to the presence of GlsR17 in the cytoplasm or background. It is possible that *Giardia* snoRNAs have a cytoplasmic component during maturation. After processing of the pre-snoRNA in the cytoplasm, the mature snoRNA may either be rapidly imported into the nucleus or processed by Dicer. Therefore, only small undetectable amounts of snoRNA may remain in the cytoplasm. The export of snoRNAs could provide an ideal method for bypassing the requirements for Drosha/Pahsa in producing pre-miRNAs.

### Argonaute and Dicer in *Giardia*

Although the proposed mechanism for production of pre-miRNAs is unique in *Giardia*, the remaining aspects of the miRNA pathway appear similar to that found in higher eukaryotes. These similarities include the requirement of Dicer for the production of mature miRNAs and an essential role of Argonaute in translational repression. In the absence of Dicer, mature miR2 and miR3 were virtually abolished. Additionally, both miR2 and miR3 are 26 nts in size, reflecting the expected size of the *Giardia* Dicer cleavage products based on its crystal structure [17]. GlAgo plays an essential role in miR2-mediated translational repression. The apparent formation of a GlAgo-miR2 complex *in vitro* and the specific binding of GlAgo to m^7^G-Sepharose suggests that it functions like a *bona fide* Argonaute by becoming incorporated into RISC with miRNA and competing for the cap with eIF4E [13], which results in the inhibition of translation initiation. When GlAgo was partially depleted from *Giardia*, translation of reporter mRNA RL-TS was increased by ∼20% with or without the exogenously introduced miR2. This indicates an inability of endogenous as well as the exogenously introduced miR2 to repress the translation of RL-TS when GlAgo was knocked down. Thus, the cytoplasmic component of the miRNA pathway in *Giardia* functions similarly to those seen in other eukaryotes.

### The Potential Role of miR2 in Regulating VSP Expression

A third of the mRNAs of annotated proteins identified to carry a potential target site for miR2 encode either VSPs or trophozoite cysteine-rich surface antigens. *Giardia* contains approximately 235–275 VSP genes in clusters of two to nine in a head to tail orientation [14]. Only a single VSP, however, is expressed on the cell surface at any given time [29]. Expression of multiple VSPs is only observed during VSP switching or excystation [29],[56]. Recently, Kulakova *et al*. showed that the activation of VSP expression is regulated by acetylation of the histone upstream of the gene [57]. It is, however, still unclear how VSP exchange occurs. During VSP exchange, expression of the currently expressed VSP must be repressed and the expression of a new VSP has to be up-regulated. In this genetic turmoil, it is possible that multiple VSP mRNAs are transcribed. The role of miR2 may be to differentially limit the translation of a particular family of 22 VSP mRNAs. It is possible that the 235–275 VSP transcripts can be classified into several families each possessing a similar 3′-UTR targeted by a specific miRNA. When the level in one of these miRNAs is lowered, a specific family of VSP will be over expressed, though it is still not clear how only one of the family members will eventually be expressed on the cell surface. The 22 identified VSP genes carry a similar miR2 target site, but they are not identical. Differences in the free energy of binding between miR2 and individual VSP target sites could differentially regulate the expression of each VSP so that only one of them eventually becomes expressed on the cell surface when the level of miR2 is lowered to a certain specific range. This may contribute to the pathogenicity of *Giardia* by limiting the number of antigens exposed to the immune system at a given time and thereby increasing the number of VSPs novel to the host. As a consequence, *Giardia* may be able to evade the host immune response. The precise role of miR2 in regulating expression of the 22 VSP genes needs to be investigated further.

### GlsR17 Localizes to Only One of the Two Nuclei in *Giardia*

There has been an accumulation of experimental data over the past years indicating that the two nuclei in *Giardia* are virtually identical. Kabnick K.S. and Peattie D.A. showed that both nuclei contain equivalent rDNA based on *in situ* hybridization and that both nuclei are transcriptionally active based on the incorporation of [^3^H] uridine [31]. Yu *et al*. used FISH and demonstrated that each of the two nuclei has a complete copy of the genome and are partitioned equally during cytokinesis [32]. Therefore, the localization of GlsR17 to the nucleoli of a single nucleus was surprising. Localization of the other identified snoRNAs will determine if this is a unique occurrence or a general phenomenon in *Giardia*.

Differences in the number of chromosomes [58] as well as discrepancies in the number of nuclear pores [59] between the two nuclei have recently been described in *Giardia*. Since the number of nuclear pores has generally been correlated to transcription activity in other eukaryotes [59], it suggests that one of the two nuclei may have higher transcriptional activities than the other. It is possible that one of the two nuclei may be solely responsible for production and export of some or all of the snoRNAs. A fascinating aspect may concern the apparent exclusive import of the snoRNAs back into the same original nucleus and the mechanism dictating it. Clarifications of this unusual finding will have to wait for further investigation.

### The Potential Evolutionary Significance in Identifying snoRNA as Pre-miRNA in *Giardia*

*Giardia* is a deeply branched eukaryote showing a combination of prokaryotic and eukaryotic features [14]. The presence of the miRNA pathway in this organism suggests that miRNA-mediated repression of translational initiation could be an ancient mechanism of gene regulation and that the snoRNAs may represent the original miRNA precursors. During evolution, snoRNAs, while maintaining their original function in ribosomal RNA maturation, may have become involved in gene regulation. These small non-coding RNAs would make ideal substrates for a primative RNase III enzyme before the evolution of new enzymes for the production of miRNAs from other much larger precursors. Since snoRNAs have been routinely discarded from the libraries of potential miRNA precursors in the past, it is not inconceivable that some of the snoRNAs in higher eukaryotes may still assume the role of miRNA precursors today, and could be readily tested.

## Materials and Methods

### Purification of *Giardia* Argonaute

The *Giardia* Argonaute was PCR amplified from *Giardia* genomic DNA using primers Ago full U-2 (5′ GAG CCC GGG TCA CTA GTG CCA TGG TAG CAG ATG TTG TCA C 3′) and Ago full L-2 (5′ GAG CTC GAG GCG GCC GCC TAG TGG TGG TGG TGG TGG TGT ATG AAG AAT GGT CTG TAC T 3′). The amplified product was cloned into the pGEM-T Easy vector (Promega), sequenced, and subcloned into the pET28b (Novagen) expression vector using *Nco*I/*Xho*I. The pET28b GlAgo was electroporated into BL21(DE3) cells containing the pG-KJE8 plasmid (Takara), which provides over-expression of chaperone proteins. An overnight culture was diluted 1∶100 into fresh LB media containing 10 ng/ml tetracycline and 4 mg/ml arabinose and incubated at 37°C for 1 hour to allow for the expression of chaperone proteins. Expression of GlAgo was induced with 0.1 mM IPTG and incubated at room temperature for 5 hours. Pelleted cells were lysed with BugBuster (Novagene) and bound to Ni-NTA beads in the presence of 40 mM imidazole, 5 mM ATP, and 10 mM MgCl_2_ at 4°C overnight. Beads were washed with 15 ml of wash buffer (20 mM sodium phosphate, pH 60; 500 mM NaCl) containing 60 mM imidazole and the protein was eluted in 4 ml of wash buffer containing 150 mM imidazole, concentrated and transferred to storage buffer.

### Small RNA Cloning

Small RNAs were cloned following the protocol from the Ambros lab (http://banjo.darthouth.edu/lab/MicroRNAs/Ambros_microRNAcloning.htm). In short, *Giardia* cells were lysed and small RNAs were enriched using the *mir*Vana kit from Ambion. The purified small RNAs were further size fractionated using the Ambion FlashPAGE fractionator to select small RNA less then 40 nts. The small RNAs were then linked to a 3′ linker (AMP-5′p-5′p/CTG TAG GCA CCA TCA AT di-deoxyC- 3′) and size fractionated on a 15% urea-polyacrylamide gel. Those with attached 3′ linkers (∼40 nt) were electroeluted from the gel and ethanol precipitated. The 3′ linked small RNAs were then ligated to a 5′ linker (5′- ATC GTrA rGrGrC rArCrC rUrGrA rArA –3′; “r” denotes RNA). This step is designed to select for small RNAs with a 5′ monophosphate, as would be expected from Dicer processing. The reaction was cleaned by phenol/chloroform extraction followed by ethanol precipitation. The cleaned product was used in an RT-PCR reaction to make cDNA, which was gel purified, digested with *Ban*I and ethanol precipitated. The cleaned product was used in a ligation reaction to concatamerize the PCR product together. The reaction was run on an agarose gel and fragments between 600–1000 bp were isolated. The isolated fragments were re-amplified using PCR and cloned into pGEM-T Easy using the pGEM-T Easy kit (Promega). Colonies containing insert were sequenced.

### Hammerhead Ribozyme Mediated Knockdown of *Giardia* Argonaute and Dicer

The Hammerhead ribozyme was incorporated into the GlAgo antisense RNA using recombinant PCR. Briefly, PCR1 containing the 5′ complementary sequence and the ribozyme was amplified using Ago HHR PCR1 F (5′ CGC GCG CTC GAG CTC CCA GAT TGA CCT GGG ATC 3′) and Ago HHR PCR1 R (5′ CTG CCC CTG AAC TAT AGA GTG CTG ATG AGT CCG TGA GGA CGA AAC TCT GAA AAC CTT TCC GTT G 3′; ribozyme underlined). PCR2 containing the 3′ complementary sequence was amplified using Ago HHR PCR2 F (5′ CAC TCT ATA GTT CAG GGG CAG 3′) and Ago HHR PCR2 R (5′ GCG CGC GAG CTC CTT CAA TGG TAA CTA TAC GAG 3′). Purified PCR1 and PCR2 were then used as the template for the recombinant PCR using Ago HHR PCR1 F and Ago HHR PCR2 R. The recombinant PCR was cloned into pGEM-T Easy and sequenced. The Hammerhead ribozyme surrounded by 500 nt of complementary GlAgo sequence was then cloned into pC631pac using the *Xho*I restriction site. This clone was linearized with *Nru*I and used for *in vitro* transcription. The transcribed RNA was transfected into giardiavirus-infected *Giardia*. Puromycin (100 µg/ml) was used to select for expression of the ribozyme.

A similar approach was used to incorporate a Hammerhead ribozyme into the Dicer antisense. PCR3 was obtained using primers Xho-Dicer 5′ (5′ GCC TCG AGT TTA GTA GGA ATG CAT GCT TTG G 3′) and Dicer RZ2 (5′ GGG TAG AAT CGA TCC CAA GAA CCT GAT GAG TCC GTG AGG ACG AAA CAT AAA GAG ACC AGC 3′; ribozyme underlined). PCR4 was obtained using primers Xho-Dicer 3′ (5′ GCC TCG AGG GAT ATT ACA CTA CGC ATC AGC 3′) and Dicer RZ1 (5′ GCT GGT CTC TTT ATG TTT CGT CCT CAC GGA CTC ATC AGG TTC TTG GGA TCG ATT CTA CCC 3′; ribozyme underlined). Purified PCR3 and PCR4 were used as templates for recombinant PCR using Xho-Dicer 5′ and Xho-Dicer 3′. After cloning into pGEM-T Easy and sequencing, the PCR product was cloned into the pC631neo vector using *Xho*I and the *in vitro* transcript transfected into giardiavirus-infected *Giardia* as described above. Neomycin (800 µg/ml) was used to select for expression of the ribozyme.

### miRNA Assay

*Giardia* WB trophozoites were grown in modified TYI-S-33 media to a density of 10^7^ per ml, washed twice in phosphate buffered saline (PBS), once in electroporation buffer (10 mM K_2_HPO_4_–KH_2_PO_4_ (pH 7.6), 25 mM HEPES (free acid), 120 mM KCl, 0.15 mM CaCl_2_, 2 mM EGTA, 5 mM MgCl_2_, 2 mM ATP, 4 mM Glutathione), and finally resuspended in electroporation buffer. RL-TS mRNA (3.5 µg), yeast tRNA (125 µg), and, if needed, 1 µg of 5′-phosphate-miR2 RNA (miR2) (IDT) were added to the cell suspension, incubated on ice for 10 minutes and then subjected to electroporation using a Bio-Rad Gene Pulser Xcell (Voltage: 450 V, Capacitance: 500 µF, Resistance: ∞). Cells were then incubated on ice for 10 minutes and added to pre-warmed culture medium. The transfected cells were incubated at 37°C for 5 hours, pelleted, washed once in PBS, and lysed using the *Renilla* luciferase assay kit (Promega). The lysate was centrifuged at 12,000 g for 2 min to remove cellular debris. The cleared lysate was used to test for *Renilla* luciferase activity. The protein concentration of the cleared lysate was measured by the Bradford method (Bio-Rad) and used to normalize the luciferase activity.

### RNA Isolation

Total RNA was isolated from *Giardia* using Trizol (Invitrogen) while RNA <200 nts was isolated using the *mir*Vana kit (Ambion). Further fractionation of total RNA to <40 nts was accomplished by standard denaturing PAGE and electroelution or by using the Ambion FlashPAGE followed by ethanol precipitation.

### Splinted Ligation

Splinted ligation was preformed as previously described [26]. Size fractionated RNA was incubated with 100 pmoles of the “bridge” oligo B1 (5′ C3 spacer-GAA TGT CAT AAG CGG GAC TAT AGG GGC GGT GAT TAG GCT G–C3 spacer 3′) containing the miR2 binding site (underlined) and 100 fmoles of the “linker” oligo L1 (5′ CGC TTA TGA CAT TCddC 3′) with 20 mM Tris-HCl (pH 8.0) and 75 mM KCl. The reaction mixture was incubated at 95°C for 1 min, 65°C for 2 min and 37°C for 10 min. Finally, 1X T4 DNA ligase buffer and 10 U of T4 DNA ligase (NEB) was added to the reaction and incubated at 30°C for 1 hour. The ligase was heat inactivated by incubation at 75°C for 15 min. The reaction mixture was loaded on to a pre-run 15% denaturing urea-polyacrylamide gel and quantified using a PhosphorImager.

### Fluorescence *in situ* Hybridization

*Giardia* WB trophozoites were harvested by placing culture tubes on ice for 15 minutes and centrifuging to pellet the cells. The cells were suspended in 1 ml of modified TYI-S-33 culture medium, placed on cover slips pretreated with 0.1% poly-L-lysine, and incubated at 37°C for 30 minutes to allow the trophozoites to adhere. They were then fixed in 4% paraformaldehyde for 30 minutes at room temperature and washed with PBS and 2X SSC (300 mM NaCl, 30 mM sodium citrate) for 5 minutes each. The cells were permeabilized with 0.5% Triton X-100 for 5 minutes at room temperature and dehydrated in 70% ethanol followed by 100% ethanol for 5 minutes each. The dehydrated cells were denatured with 2X SSC in 70% formamide for 2 minutes at 70°C and dehydrated again with cold 70% and 100% ethanol. Salmon DNA (10 µg), yeast tRNA (25 µg), and 100 pmoles of the GlsR17 RNA 5′-UTR probe (5′ 6-carboxyfluorescein (FAM)–CCC GGA UCC UCA CCA CGA GUA AAC CC 3′) and miR2 RNA 3′-UTR probe (5′ Cy3–GGA CUA UAG GGG CGG UGA UUA GGC UG 3′) (IDT) were added to 100 µl of 100% formamide, heated at 75°C for 10 minutes and placed immediately on ice. The probe was mixed with an equal volume of hybridization buffer (4X SSC, 20% dextran sulfate, and 4 mg/ml BSA), added to the cover slip and incubated overnight at 37°C. The cover slips were washed 3 times with 0.1% SSC in 50% formamide at 50°C for 5 minutes each to remove unhybridized probe, placed facedown on clean glass slides with 1 drop of Vectashield (Vector Labs) mounting media with DAPI (4′,6 diamidino-2-phenylindole) and sealed with paraffin wax. Cells were examined using a Nikon TE2000E motorized inverted microscope equipped with 60X bright-field and epifluorescence optics. Images were acquired with the NIS-Elements Advanced Research software (Nikon) and analyzed with ImageJ (http://rsbweb.nih.gov/ij/index.html).

## Supporting Information

Figure S1 Effects of ribozyme-mediated knockdowns of *Giardia* Argonaute and Dicer gene expression on the growth of *Giardia* trophozoites. Semi-quantative RT-PCR was performed to monitor the decrease of GlAgo and Dicer mRNA with α-2-giardin mRNA and α-tubulin mRNA included as sampling controls, respectively.
(1.74 MB TIF)

Figure S2 Presence of a cap-binding motif in GlAgo. Recombinant His-tagged GlAgo from transformed *E. coli* was incubated with m^7^GpppG-Sepharose in binding buffer (20 mM HEPES, pH 7.4; 150 mM KCl; 1 mM EDTA; 2 mM dithiothreitol). The total input and flowthrough are shown in lanes 1 and 2. The beads were washed with 10 column volumes of binding buffer (lanes 3 and 4), which was followed by 10 more column volumes of binding buffer containing 0.1 mM GTP to remove any non-specific binding (lanes 5 and 6). The remaining protein was eluted with 0.1 mM m^7^GpppG. Collected fractions were analyzed by SDS-PAGE followed by Western blot probed with an anti-His antibody.
(7.66 MB TIF)

Figure S3 MFOLD analysis of full-length GlsR17. Minimum energy structures of GlsR17 show the formation of hairpin loop structures in an H/ACA type fold. The mature miR2 (red), Box C (blue), and Box D (green) motifs are shown.
(2.96 MB TIF)

Figure S4 Gel shift analysis of GlAgo/miR2 interaction. Purified recombinant GlAgo was incubated with 10 ng of radiolabeled miR2 and analyzed in native 6% polyacrylamide gel electrophoresis. Increasing concentrations of GlAgo resulted in an increased amount of radioactivity in gel shift. This interaction can be competed off with unlabeled miR2 or yeast RNA.
(3.95 MB TIF)
