# Supplementary figures and images for: snoRNA, a Novel Precursor of microRNA in Giardia lamblia
Source: PLoS Pathog. 2008 Nov 28;4(11):e1000224. doi: 10.1371/journal.ppat.1000224 (PMC2583053; doi:10.1371/journal.ppat.1000224)

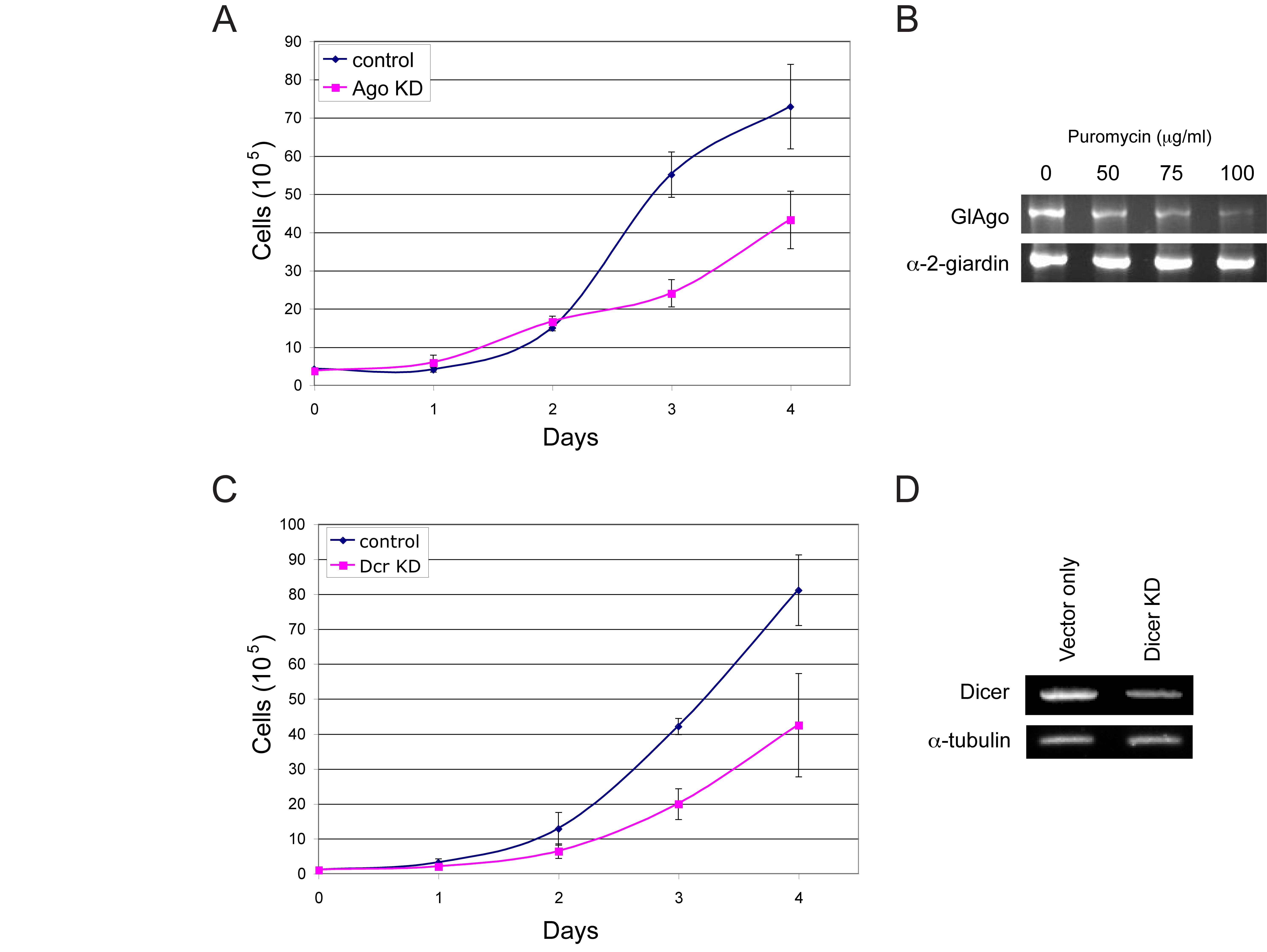

Supplement: Figure S1 — Effects of ribozyme-mediated knockdowns of Giardia Argonaute and Dicer gene expression on the growth of Giardia trophozoites. Semi-quantative RT-PCR was performed to monitor the decrease of GlAgo and Dicer mRNA with α-2-giardin mRNA and α-tubulin mRNA included as sampling controls, respectively. (1.74 MB TIF) [file ppat.1000224.s001.tif]

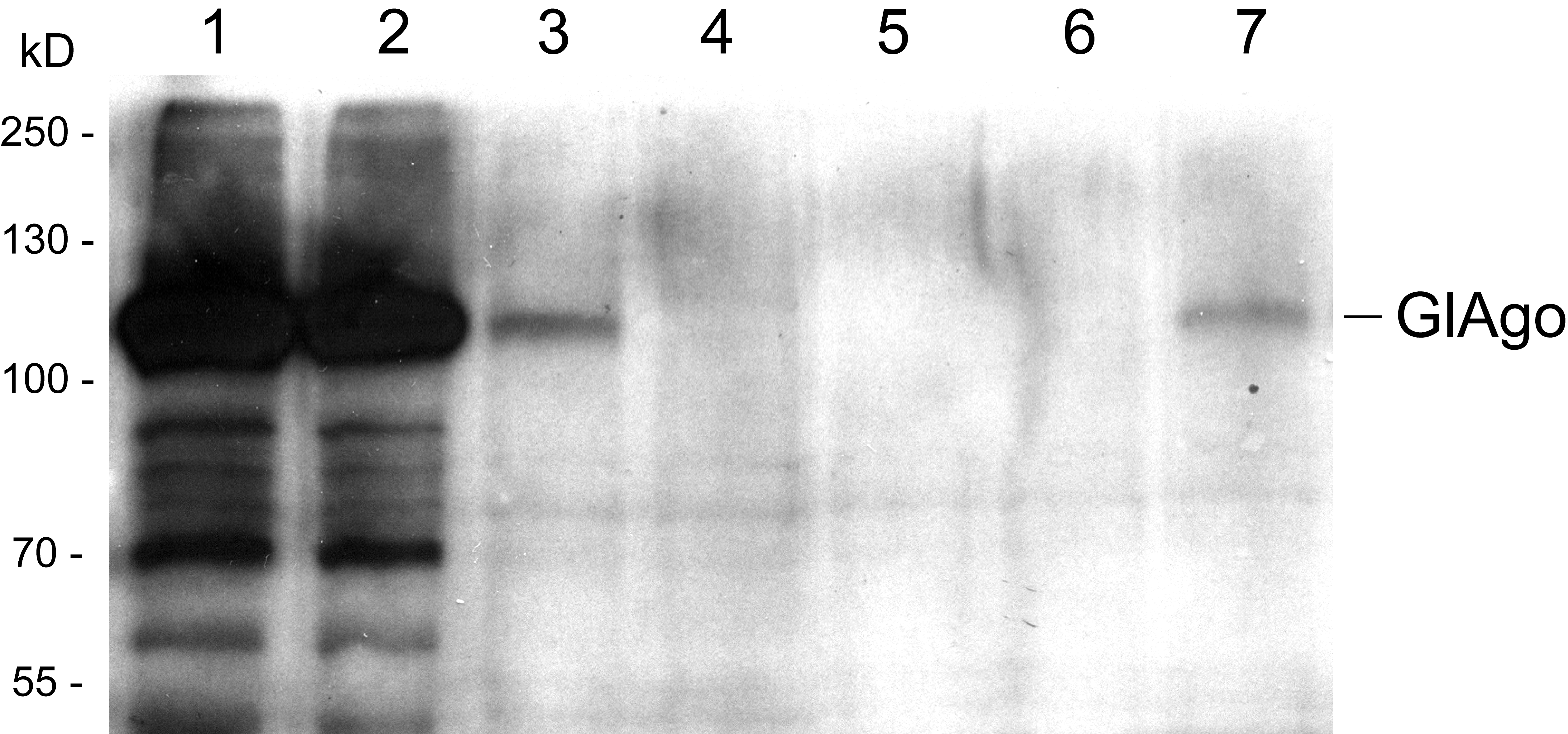

Supplement: Figure S2 — Presence of a cap-binding motif in GlAgo. Recombinant His-tagged GlAgo from transformed E. coli was incubated with m7GpppG-Sepharose in binding buffer (20 mM HEPES, pH 7.4; 150 mM KCl; 1 mM EDTA; 2 mM dithiothreitol). The total input and flowthrough are shown in lanes 1 and 2. The beads were washed with 10 column volumes of binding buffer (lanes 3 and 4), which was followed by 10 more column volumes of binding buffer containing 0.1 mM GTP to remove any non-specific binding (lanes 5 and 6). The remaining protein was eluted with 0.1 mM m7GpppG. Collected fractions were analyzed by SDS-PAGE followed by Western blot probed with an anti-His antibody. (7.66 MB TIF) [file ppat.1000224.s002.tif]

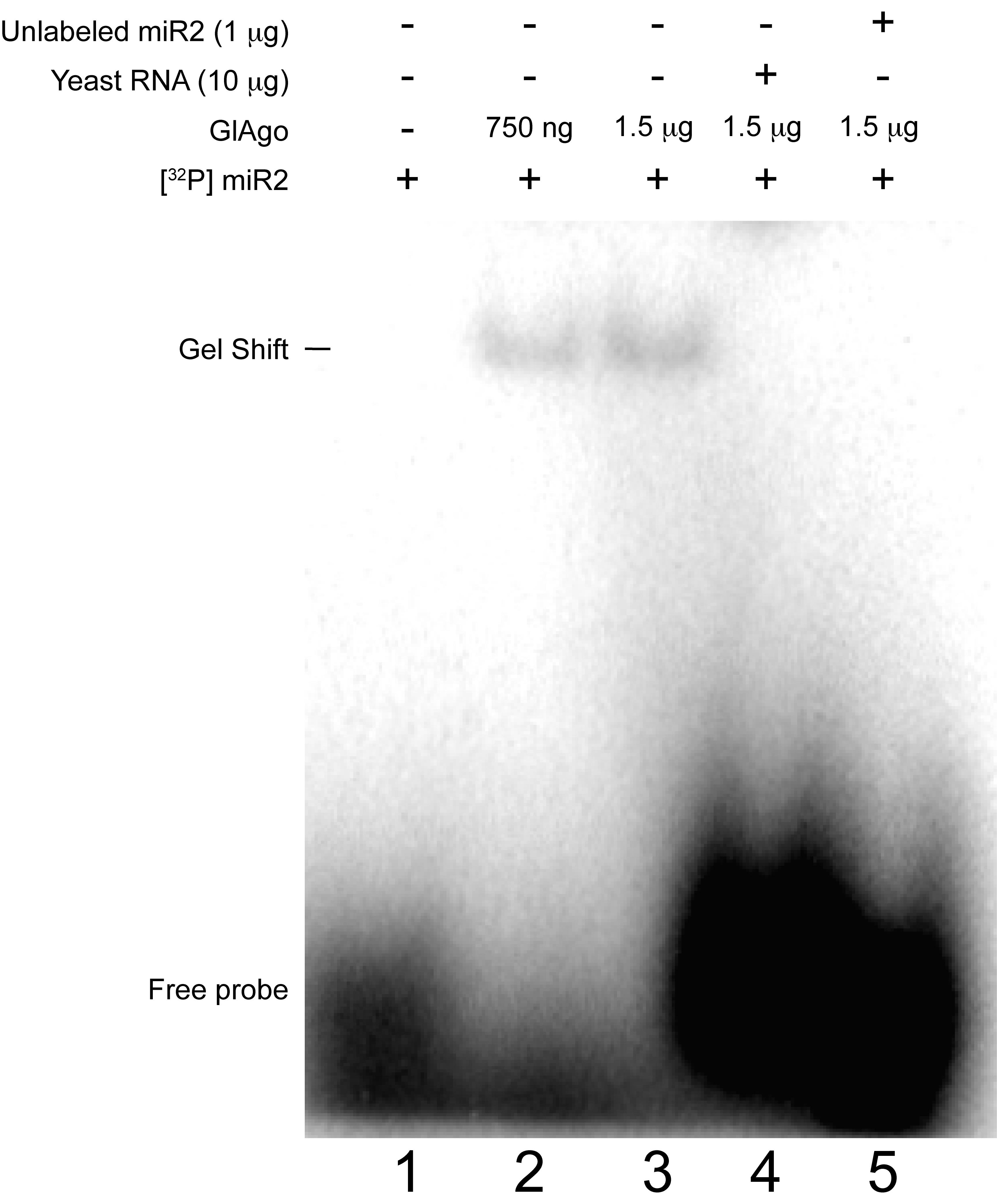

Supplement: Figure S4 — Gel shift analysis of GlAgo/miR2 interaction. Purified recombinant GlAgo was incubated with 10 ng of radiolabeled miR2 and analyzed in native 6% polyacrylamide gel electrophoresis. Increasing concentrations of GlAgo resulted in an increased amount of radioactivity in gel shift. This interaction can be competed off with unlabeled miR2 or yeast RNA. (3.95 MB TIF) [file ppat.1000224.s004.tif]
